# Online Group Course for Parents With Mental Illness: Development and Pilot Study

**DOI:** 10.2196/jmir.1394

**Published:** 2010-12-19

**Authors:** Rianne AP van der Zanden, Paula AM Speetjens, Karlijn SE Arntz, Simone A Onrust

**Affiliations:** ^1^Trimbos InstituteNetherlands Institute of Mental Health and AddictionUtrechtNetherlands

**Keywords:** Parenting support, health promotion, parents with mental illness, online group course, Internet

## Abstract

**Background:**

Children of parents with mental illness (COPMI) are at greater risk of developing mental disorders themselves. Since impaired parenting skills appear to be a crucial factor, we developed a facilitated 8-session preventative group course called KopOpOuders (Chin Up, Parents) delivered via the Internet to Dutch parents with psychiatric problems. The goal was to promote children’s well-being by strengthening children’s protective factors via their parents. To reach parents at an early stage of their parenting difficulties, the course is easily accessible online. The course is delivered in a secure chat room, and participation is anonymous.

**Objective:**

This paper reports on (1) the design and method of this online group course and (2) the results of a pilot study that assessed parenting skills, parental sense of competence, child well-being, and course satisfaction.

**Method:**

The pilot study had a pre/post design. Parenting skills were assessed using Laxness and Overreactivity subscales of the Parenting Scale (PS). Sense of parenting competence was measured with the Ouderlijke Opvattingen over Opvoeding (OOO) questionnaire, a Dutch scale assessing parental perceptions of parenting using the Feelings of Incompetence and Feelings of Competence subscales. Child well-being was assessed with the total problem score, Emotional Problems, and Hyperactivity subscales of the Strengths and Difficulties Questionnaire (SDQ). Paired samples *t* tests were performed, and Cohen’s *d* was used to determine effect sizes. Intention-to-treat analyses and analyses of completers only were both performed. Course satisfaction was evaluated using custom-designed questionnaires.

**Results:**

The sample comprised 48 parents with mental illness. The response rate was 100% (48/48) at pretest and 58% (28/48) at posttest. Significant improvements were found on PS Laxness and Overreactivity subscales (*P* < .01) and on the OOO Feelings of Incompetence and Competence subscales (*P* < .01) in analysis of completers only as well as by intention-to-treat analysis. Effects were moderate on the PS (*d =* .52 and *d =* .48) and were large and moderate on the OOO (*d* = 0.61 and *d =* 0.46). At pretest, 75% and 64% of PS scores were in the clinical range, which declined to 43% and 39% at posttest. No significant changes were found for child well-being. Scores for approximately two thirds of children were not in the clinical range at both pretest and posttest. The mean course satisfaction score was 7.8 on a 10-point scale. Of all participants, 20% (10/48) followed all the sessions.

**Conclusion:**

This online group course on parenting skills is innovative in the field of e-support and among interventions for mentally ill parents. The pilot results are promising, showing moderate to large effects for parenting skills and parental sense of competence. Test scores at baseline indicating parenting problems were largely in the clinical range, and baseline scores indicating problems among the children were in the nonclinical range, suggesting that parents were reached at an early stage. Course satisfaction was high. Future research should focus on cost effectiveness and course adherence.

## Introduction

Parenting is a complex social skill, and it can be heavily undermined by mental illness [1]. Parental mental illness is a widespread phenomenon. One in four to five adults experience mental health problems at some stage of their lives [2,3], and a considerable proportion of them are bringing up children at the time. Every year in the Netherlands, 864,000 parents suffer psychopathology according to Diagnostic and Statistical Manual of Mental Disorders (DSM) criteria, and together they have 1.6 million children under 22 years of age [4,5]. Children of parents with mental illness are themselves at risk of developing mental disorders such as depression, anxiety, and alcohol or drug dependence [5]. They are more likely than other children to have poorer communication skills and relationship or intimacy problems [6-11]. The risks are substantial: children who have a parent with psychiatric problems are 1.5 times more likely to develop a mental disorder at some point in their lives than children without such parents (50% versus 30%), and as many as 66% develop disorders if both parents are mentally ill [4,5]. Despite this risk, a considerable number of children do not develop disorders, indicating that parental psychopathology alone does not explain the problem. The precise mechanisms through which children develop mental health problems are still unknown, but a combination of genetic, biological, social, and psychological risk and protective factors is generally assumed to be the cause [12].

### Risk Factors

The presence of risk factors in children of parents with mental illness is associated with an increased probability of onset of major health problems as well as greater severity or longer duration of these problems [13]. Knowledge of risk factors is crucial to illness prevention programs, as some factors can be alleviated or eliminated. Well-known risk factors that cannot be influenced directly by preventive intervention are the child’s age at the onset of a parental disorder [13], genetic factors [14,15], and the severity and duration of parental illness [16]. Yet the medical literature has also drawn attention to several types of risk factors that can be mitigated by preventive intervention. These are described below.

### Dysfunctional Parent-Child Interaction

Parents with mental illness interact differently with their children than other parents. The parenting styles of mothers with unipolar depression, for instance, may be characterized by a flatter affect and less physical contact, lower levels of expressed approval or spontaneity, and more frequent anger [9,17,18]. Anxious parents exhibit high levels of control, disapproval, and overprotection towards their children [14,19]. Alcohol-dependent parents often show neglect and unpredictable behaviors [6,20]. A further danger is the increased risk of child abuse by parents with mental illness [20,21]. Parents may put age-inappropriate responsibilities on children, resulting in “parentification” of the child [22-24]. In addition to behaviors and symptoms stemming from their mental illness, parents also experience feelings of shame and fears of losing custody of their children; these may also negatively affect parent-child interaction and may inhibit parents from seeking help [25,26].

### Conflicts Between Parents

In addition to the parents’ individual problems, there may be problem-related conflicts between parents, for instance conflicts about an alcoholic parent’s drinking. Parental stress and conflicts show associations with undue pressure and disapproval exerted on children [27]. Conflicts and stress can have a negative impact on the children [11].

### Partners and Lone Parents

A mental disorder in one parent can put growing pressure on the well partner. If the partner can meet the challenge, the consequences for the family and the children may remain limited [28]. Growing up in a single-parent family is in itself a considerable risk factor to children for developing mental disorders, and the combination with parental mental health problems adds extra weight [29].

### Protective Factors

From the point of view of mental illness prevention, protective factors are at least as important as risk factors. Protective factors are conditions that improve an individual’s resistance to risk factors and illness; they have been defined as “those factors that modify, ameliorate, or alter a person’s response to some environmental hazard that predisposes to a maladaptive outcome” [13]. Although the evidence base on protective factors is still limited [6], the following factors have been identified in the literature:

- If a parent and child have a good relationship despite the parental disorder, the child’s prognosis is significantly improved [23,24].
- Strong support of the child by the unaffected parent may compensate for a deficit in support from the affected parent. In broader terms, a good relationship with at least one parent is a strong protective factor: a child can then cope with considerable difficulties without necessarily developing psychopathology. Social support from the unaffected parent, a sibling, or a support network or trusted person outside the family can help protect the child. Emotional and practical support are both important [23,30-32].
- Realistic self-appraisal on the child’s part is crucial [30,33,34].
- A clear understanding of the parent’s problems can be very helpful [4,23].

### Parenting Support Programs

According to a study by Goodman and Brumley [35] that compared depressed (n = 25), schizophrenic (n = 53), and well mothers (n = 23), parenting style is a decisive factor for children’s outcomes. The study showed that effects of a mother’s illness on a child are mediated mainly through the quality of parenting as she practices it. Affectional involvement and parental responsiveness are particularly important for a child’s social functioning.

Parenting style can be improved by parenting support programs. Many studies have shown that preventive parenting support has positive effects on parents’ skills and sense of parental competence as well as on child well-being [36-41]. A well-known parenting program is the Triple P Positive Parenting Program. Level 4 of this program is indicated if the child has multiple behavior problems in a variety of settings and there are clear deficits in parenting skills. A meta-analysis examining the effects of level 4 of Triple P showed a moderate effect on the Parenting Scale (overall effect size *d* = 0.51) and a large effect on the Parenting Sense of Competence Scale (overall effect size *d* = 0.67). These are validated scales measuring dysfunctional discipline styles and parents' view of their competence as parents. [40]. In addition, moderate positive effects were found on the Behavior Problem scale as measured by the Eyberg Child Behavior Inventory (overall effect size *d* = 0.42) [41].

### Parenting Support Programs for Parents With Mental Illness

Evidence-based parenting programs for parents with mental disorders are less common [12,42]. Also, the parental role remains an underexposed issue in the field of mental health treatment [43] even though it constitutes a fundamental part of a parent’s identity. There are indications that parents’ recovery from mental illness can be facilitated by a strengthening of their parental role [44].

According to a review by Fraser and colleagues [12], one of the few well studied, effective programs for parents with mental illness is the Preventive Family Intervention (FPI) from the United States [45], an intensive program that includes home visits to parents with mood disorders and their families. It is designed to improve communication about the disorder and its consequences for the children as well as to strengthen the children’s resilience. A study [45] of 36 families that had a nondepressed child between the ages of 8 and 15 and a parent who had experienced affective disorder were randomly assigned to either the FPI intervention or a lecture discussion group. Children in the FPI group reported significantly greater understanding of parental affective disorder. Furthermore, children and parents had significantly better adaptive functioning in terms of changes in illness-related behaviors and attitudes (eg, increased communication with and understanding of the children). This intervention has also been implemented in the Netherlands.

Less intensive parenting support programs that are easily accessible and can reach parents at an early stage of parenting problems and children’s problems are not yet available for parents with mental illness. The preventative intervention KopOpOuders (Chin Up, Parents), an online group course, is intended to fill this gap. The advantages of an online group intervention for this target group are that it is anonymous (important because participants may feel shame or may fear losing custody of their children), requires no traveling time or babysitter, and enables contact with other parents in similar situations. KopOpOuders may also reach parents who are not in touch with mental health services.

The KopOpOuders intervention is an innovative intervention in several ways. Online group courses are still rare in the entire field of e-support. Only two online studies have been reported worldwide [46,47]. These have involved chat room courses for adolescents with internalizing problems, and they have been associated with favorable effects using a pretest-posttest design. In one of the few studies of online parenting support, Taylor and colleagues [48] reported that a computer-based course combined with home visits and telephone coaching was associated with positive outcomes. Of a total of 128 goals set by 90 participants, 100% progress was made on 68 goals. Adherence to the program, which has been flagged as a potential disadvantage of e-interventions [46,47,49], was acceptable, with two thirds of participants completing all program elements. Finally, in the field of interventions for mentally ill parents, KopOpOuders is innovative because it is based on systematic evidence of risk and protective factors and parenting support theories.

### Objective

This paper describes the design and method of the online group course KopOpOuders and reports on the results of a pilot study that assessed parenting skills, parental sense of competence, child well-being, and course satisfaction.

## Methods

### The Parenting Support Course KopOpOuders.nl

The purpose of the KopOpOuders intervention for parents with mental illness is to enhance their children’s psychosocial well-being and to protect the children from developing mental health problems by improving their parents’ skills. We based KopOpOuders on recognized theories relevant to parenting support—social learning theory [50], the theory of developmental psychopathology [51], and the contextual theory [52]—and we linked course components to the identified risk and protective factors. This is consistent with Fraser's [12] call to develop theory-based interventions for this target group. As our central focus was on the risk and protective factors for children that can be influenced by giving parenting support to their parents, we chose the following focal points for the preventative intervention [4,22-24,34]:

- strengthening parent-child interaction
- supporting the unaffected parent
- ensuring a support network or trusted person for each child
- reinforcing children’s coping and social skills
- explaining the parental mental illness to the children.

Textbox 1 shows how we operationally defined these focal points.

**Objectives of the online KopOpOuders course and their operational definitions**

**(1) Good parent-child interaction**

- Parent feels less guilt and shame about the mental illness and about the consequences for the home situation.
- Parent knows what effects their own mental illness could have on the children.
- Parent knows which protective factors exist for the child and is able to strengthen these.
- Parent can articulate their own limitations and needs with respect to their parental role and can discuss these with a partner or trusted person.
- Parent learns general parenting skills (eg, setting limits, dealing with conflicts), puts these into practice, and has a realistic idea of “good-enough parenting.”

**(2) Support from the well parent**

- Well parent feels less guilt and shame about the problems in the family.
- Well parent knows how to keep functioning well and cope with the situation.
- Well parent knows partner’s limitations and needs with respect to the parental role and can discuss these with the partner.
- Well parent is able to support the partner in actively improving the partner’s parental role.

**(3) Support network or trusted person**

- Parent knows his or her own support network and enlists its help when needed.
- Parent allows children to seek support from others.
- Parent has “emergency plan” in case of relapse.
- Parent is familiar with services available to self, partner, and children and knows how to seek help there if needed.

**(4) Children’s coping skills and social competence**

- Parent knows the children’s age-specific development tasks and gives them sufficient room to perform them.
- Parent allows children to seek support from others.
- Parent informs children in age-appropriate ways about the mental illness and absolves them of responsibility.
- Parent gives children room to express their feelings.
- Parent is familiar with available services for children and knows how to seek help from them if necessary.

**(5) Children’s understanding of themselves and of the parental problems**

- Parent informs children in age-appropriate ways about the mental illness and absolves them of responsibility.

The KopOpOuders course is based on three mutually supportive principles. First, it facilitates the parents’ learning potential by highlighting and addressing their shame and guilt about their illness. Second, it teaches some general principles of parenting as well as more specific skills needed in the unique situation in which the parents and children find themselves. Third, the participants practice and consolidate this knowledge. Chat sessions, videos, and home exercises are provided to support participants as they put into practice parenting skills such as talking to children about psychological or addiction problems, listening to children, and setting limits. Consolidating the knowledge is facilitated by having participants record what they learn in a “plan of action” and fill in a “parenting atmosphere meter” every day.

The online course consisted of eight 90-minute weekly sessions in a secured chat room facilitated by one or two trained health promotion workers from four Dutch mental health organizations. (If the facilitator was highly experienced, one was sufficient.) Each course group had a maximum of six participants with mental illness. Between sessions, parents did homework and practiced parenting skills in structured home exercises. Participants were encouraged to invite their partners to read the session transcripts on the screen and to help carry out the homework exercises. The course focal points listed in Textbox 1 were addressed systematically in the eight sessions. Session topics were as follows: (1) getting acquainted and discussing the family situations; (2) “good-enough parenting”; (3) communicating with your child; (4) child development and “parentification”; (5) giving attention to your child; (6) setting limits and dealing with conflicts; (7) social network and emergency plan; (8) preserving your gains and farewell.

The chat room in which the course was delivered was part of the public website www.kopopouders.nl, which provided written information and videos about mental illness and parenting, a user forum, and an email service through which users could get individual support from a health promotion professional. The secured chat room screen had two parts: the left part was for chatting, and in the right part, the facilitator could post short videos to enhance recognition or other information such as the session agenda or an outline or diagram. The chat room screen included emoticons that participants could use to add a feeling to a message. To sign up for the course, participants completed online questionnaires. When accepted, participants received a log-in code. Registration was anonymous, but participants were asked to supply a mobile phone number to which an automatic text reminder could be sent half an hour before each weekly session.

### Sample

From March 3, 2008, through May 13, 2009, 94 parents with mental illness, 88% of them female, enrolled in the KopOpOuders program. The parent's average age was 37 years with a range of 25 to 52 years (SD 6.8), and their children’s average age was 7.7 years, with a range of 1 to 21 years (SD 4.8). Accepted for the intervention were 85 parents with mental illness; 6 others did not respond further after completing the initial questionnaires, and 3 were excluded because of the longtime placement of the child out of the home, there were no parental psychological problems, or the children were over 21 years of age. Of those parents accepted, 26 withdrew before the course started citing reasons that included an unstable home situation (divorce, relocation, starting a rehabilitation, training, or reintegration program), postponement of participation, or no reason. Ultimately, 59 parents with mental illness began the intervention, 48 of whom gave informed consent to take part in the pilot study. The reasons that 11 parents failed to provide consent are unknown, but these parents were all female, 8 (73%) lived in single-parent families, 11 (73%) had intermediate or lower vocational education, and most reported that they experienced a mood disorder or a borderline personality disorder.

In the informed consent group (n = 48), 41 (85%) participants were female with a mean age of 37 years (SD 6.8); the mean age of their children was 6.7 years (SD 5.3). The following mental health problems were reported: depression or bipolar disorder (41%), personality disorder (38%), post-traumatic stress disorder (19%), attention-deficit/hyperactivity disorder (8%), anxiety disorders (6%), psychosis (6%), eating disorders (4%), alcohol addiction (4%), and autism (2%). Comorbidity was reported by 33% (16/48). Of the 48 participants, 28 (58%) lived in two-parent families or stepfamilies, 40 (83%) had one or two children, 27 (56%) were married, 43 (90%) were of Dutch ethnicity (the others were Belgian, Turkish, and Danish), 20 (42%) had intermediate and 13 (27%) higher vocational education, 25 (52%) had jobs or attended reintegration programs, and 45 (93%) had received professional psychological help from a mental health service. The partners of the mentally ill parents were not involved in the study.

### Recruitment and Screening

Parents with mental health problems were recruited via the website, www.kopopouders.nl, or through recruitment materials distributed by four implementing mental health agencies, both internally, and to other mental health services in their regions. These included general practitioners, social services, and homecare services in four rural and urban regions in the Netherlands. Parents applied for the course via the website by completing questionnaires about their childrearing situation and the nature of the problems. Parents accepted for the course were also asked for their consent to take part in the study. Exclusion criteria for course acceptance were long-term placement of the children out of the home, severe personality or behavioral problems of children, the presence of acute crisis situations, and insufficient proficiency in Dutch.

### Measures and Design

At the start of the course (at pretest), parents completed questionnaires on parenting practices, child behavior, and sociodemographic background. The course comprised eight 1.5-hour sessions. At the end of the eighth session (posttest), participants completed questionnaires on course satisfaction, parenting practices, and child behavior.

#### Parenting Skills

To assess parenting practices, we used 12 questions from the Laxness and Overreactivity subscales of the Dutch version of the Parenting Scale (PS) [53-55]. These scales measure the parenting style during the last two months on a 7-point scale. Subscale Laxness measures the degree in which parents apply a permissive parenting style. An example of the questions in the subscale Laxness is: “When I say my child can’t do something…I let my child do it anyway.” The response choices range from 0, “never or rarely” to 7, “I stick to what I said.” The subscale Overreactivity measures the degree of authoritarian parenting style and a parent's appropriate reaction to child behavior. An example is: “When my child misbehaves, I spank, slap, grab, or hit my child.” The response choices range from 0, “never or rarely” to 7, “most of the time.” Both subscales consist of 6 items. The corresponding scores sum up to a total subscale score with a range of 6 to 42. A low score means use of effective parenting skills, and a high score indicates a dysfunctional parenting style. Both subscales, Laxness and Overreactivity, tested as reliable with Cronbach alpha (Laxness .79; Overreactivity .88). The original Parenting Scale in English included an additional subscale, Verbosity, intended to measure the degree of verbalization of parenting reactions. The internal consistency of this subscale has been found to be unsatisfactory [56-59]. Therefore, this subscale was not included in the study. Without including a score for the subscale Verbosity, the total problem score in the short Dutch PS is not reliable [54,55]. Therefore, the total score was not included in the analyses. The parenting scale has cutoff scores that can be used for a clinical assessment of dysfunctional parenting. For Laxness, the clinical cutoff score is 2.8 and higher, and for Overreactivity the cutoff score is 3.0 and higher [54,55]. The percentage of parents with scores within the clinical range of the questionnaire is reported in the “Results” section.

#### Parental Competence

Sense of parental competence was measured with a Dutch scale assessing parental perceptions of parenting: the Ouderlijke Opvattingen over Opvoeding questionnaire (OOO). The OOO has 11 questions that can be divided into two subscales: Feelings of Incompetence (6 items) and Feelings of Competence (5 items). Answers are rated on a 6-point scale with categories ranging from 1, “completely disagree” to 6, “completely agree.” The 6-item Incompetence scale of the OOO is a subscale taken from the Nijmegen Parenting Stress Index, short version [60]. The scores for this subscale range from 6 to 36; the higher the score, the more incompetent a parent feels. An example is: “Parenting my child is more difficult than I thought it would be.” The 5-item Competence scale of the OOO is taken from the Parenting Self-Agency Measure [61]. The scores range from 6 to 30; the higher the score, the more competent a parent feels. An example is: “I feel confident in my role as parent.” Both OOO subscales were reliable (Incompetence, alpha = .79; Competence, alpha = .74). There are no clinical cutoff scores available for this questionnaire.

#### Child Behavior

The official Dutch 25-item Strengths and Difficulties Questionnaire (SDQ) [62-65] was used to assess child behavior. It has 5 subscales and a total of 25 items each of which can be rated 0, “not rue,” 1, “partly true,” or 3, “true.” Subscales are Emotional Problems Scale, Behavior Problems, Hyperactivity, Peer Problems, and a Pro Social Behavior scale. Range of scores per subscale are 0 to 10, and the range of the total problem score is 0 to 40 (Pro Social scale is not included in the Total Problem score.) A higher score indicates more problem behaviors except for subscale Pro Social Behavior where a higher score means less problem behavior. The first subscale measures the emotional problems of the child. An example item from this subscale is, “My child often complains of headaches, stomach aches.” The clinical cutoff score is 5, meaning that scores above 5 indicate clinical problems, in this case abnormal emotional problems, which may be an indication for professional intervention. The second subscale measures the conduct problems of the child. An example item of this subscale is, “My child often has temper tantrums or hot tempers.” The clinical cutoff score for this scale is 4. The third subscale measures hyperactivity. An example item of this subscale is, “My child is restless, overactive, cannot stay still for long.” The clinical cutoff score for this scale is 7. The fourth subscale measures peer problems. An example item of this subscale is, “My child has at least one good friend.” The clinical cutoff score is 7. The final subscale measures pro social behavior. An example item of this subscale is, “My child is considerate of other people's feelings.” The clinical cutoff score is 4, and in the case of this subscale, scores of 4 and below indicate that there are abnormal social behavior problems that may be an indication for professional intervention. Finally, the total problem score has a clinical cutoff score of 14, meaning that scores above 14 indicate abnormal emotional and behavior problems [66]. Two of the subscales, Emotional Problems and Hyperactivity, and the total problem score were reliable in the present study. Cronbach alphas for these scales were .86, .72, and .79, respectively. Cronbach alphas for the subscales Behavioral Problems, Peer Problems, and Pro Social Behavior were .33, .43, and .55, respectively, were considered not reliable, and were omitted from further analysis. The SDQ has Dutch clinical cutoff scores presented above, and the percentage of scores within the clinical range of the questionnaire are reported in the “Results” section.

#### Family Background

A sociodemographic questionnaire gathered background data on the participants, such as family features, socioeconomic status, work status, number of children, problems within the family, and the motives for taking the course. An example item was, “What describes your family best?” The choices were: (1) regular family, that is, both parents are biological or adoptive parents; (2) stepfamily, that is, two parents one of whom is a stepparent; (3) single-parent family; (4) other.

#### Course Satisfaction

Participants’ overall satisfaction with the intervention was measured at posttest on a 10-point scale using a custom-designed evaluation questionnaire in which 10 represented highly satisfied and 1 represented highly dissatisfied. Satisfaction was also evaluated through questions on course techniques, organization, and content. Example items were, “Did you encounter technical problems using the chat room?” “How satisfied are you with the content of session 1, session 2, and so on.” Items were rated from 1 to 10.

### Statistical Analyses

KopOpOuders is an innovative e-parenting support intervention designed for a specific group of parents. Though negative results were not expected, two-sided paired samples *t* tests were conducted for conservative reasons. These results from a completer analysis of completers (ie, participants who completed the course) are reported in Table 1. We also conducted analyses based on the intention-to-treat-principle, using the conservative “last observation carried forward” method in which missing values at posttest were replaced by the value at pretest, in addition to an analysis using regression imputation where all missing values were imputed. The later was implemented in Stata version 9.4 [67]. Effect sizes were calculated as Cohen’s *d* that is, mean at pretest minus mean at follow-up divided by the standard deviation (SD) at pretest. Values of *d* less than 0.32 were interpreted as small effect sizes, values from 0.33 to 0.55 as moderate, and values from 0.56 to 1.20 as large [68,69]. Using the SD at pretest to divide the calculated difference between the pre and post mean score is more conservative than using the mean pre/post SD. Descriptive statistics were used to determine how satisfied participants were with the content and design of the course. Responses to open questions on the evaluation forms were coded as quantitative data.

## Results

### Response Rate

The sample comprised 48 parents with mental illness who consented to study participation and took part in one or more course sessions. Response rate at pretest was 100% (48/48) and at posttest 58% (28/48). A logistic regression analyses with dropout at posttest as the dependent variable was executed. Participants who completed the course did not significantly differ from participants who dropped out of the study on any of the measured variables.

**Table 1:** Short-term intervention effects on parenting skills, sense of parenting competence as measured by Ouderlijke Opvattingen over Opvoeding (parental beliefs about parenting-questionnaire) and child well-being as measured by the Strengths and Difficulties Questionnaire (n = 28)

		At Pretest			At Posttest			*t* test		
Test and Subscale		Mean	SD	Scores in the Clinical Range %	Mean	SD	Scores in the Clinical Range %	*t*	*P* Value	d
**Parenting skills**										
	Laxness	3.41	1.08	75%	2.85	0.79	43%	2.90^a^	.007	0.52
	Overreactivity	3.71	1.56	64%	2.97	1.12	39%	4.02^a^	.000	0.48
**OOO**										
	Feelings of incompetence	26.32	5.62		22.88	5.01		3.13^a^	.004	0.61
	Feelings of competence	18.57	4.66		20.70	3.69		2.81^a,b^	.009	0.46
**SDQ**										
	Emotional problems	4.36	3.08	36%	3.75	2.61	32%	1.57	.13	
	Hyperactivity	5.75	2.50	39%	5.01	2.97	25%	1.97	.06	
	Total problems	15.11	6.20	36%	13.67	6.91	36%	1.88	.07	

^a^ *P* < .01

^b^ The negative result is consistent with prediction and represents a positive change since parents feel more competent.

### Effects on Parenting Behavior

Table 1 summarizes the results of the completers-only analyses. The parents’ laxness and overreactivity ratings decreased significantly in the course of the intervention; effect sizes were moderate (*d* = 0.52 and *d =* 0.48). The Dutch Parenting Scale defines clinical cutoff scores; the percentages of parents scoring in the clinical range declined from 75% to 43% for laxness and from 64% to 39% for overreactivity. Feelings of parenting incompetence also diminished significantly from pretest to posttest, reflecting a large effect (*d =* 0.61). Feelings of competence grew and showed a moderate effect size (*d =* 0.46). The negative *t* test value is consistent with predictions of increased competence of parents. No clinical range has been defined for the OOO scale. Both intention-to-treat analyses (with missing values imputed according to the “last observation carried forward” and “regression imputation” methods) confirmed the outcomes of the reported completers-only-analyses.

### Effects on Child Behavior 

Results of the completers-only analyses are presented in Table 1. Parental reports indicated some trends in effects on their children’s behavior. Hyperactivity and total problems declined but not significantly (*P* < .10); effect sizes were small (*d* = 0.30 and *d* = 0.23). No significant effects were found for emotional problems. The results from the intention-to-treat analyses were consistent. Slightly over one-third of children had scored in the clinical range on each SDQ subscale at pretest, and this remained unchanged at posttest except for a nonsignificant decrease on the hyperactivity subscale.

### Course Satisfaction

The course satisfaction questionnaire was completed by 27 parents at the end of the intervention. Their overall mean satisfaction rate was 7.8 on a 10-point scale. The highest-rated course topic was “giving better attention to your child” (mean score 8.0). Most parents indicated that the intervention had met their expectations well. The best-met expectation was “learning to deal better with feelings of shame, guilt, and incompetence,” which was cited by 100% (14/14), followed by “finding sympathy and recognition by sharing experiences,” cited by 73% (11/15) of parents. Satisfaction with the course facilitators was high: 78% (21/27) of parents found facilitators involved and supportive. A large majority of parents, 74% (20/27), considered the online intervention a better way to receive professional help than a face-to-face intervention. Most parents, 70% (19/27), responded that they would definitely recommend the intervention to other parents. Satisfaction was also expressed with the anonymity of the course, the opportunity to participate without leaving home, and the fact that no child care was needed. Most parents, 78% (21/27), were satisfied with the duration of the sessions, and 89% (24/27) with the interval between them; 52% (14/27), were satisfied with the number of sessions, but 44% (12/27) would have preferred more sessions. The course homework, including the practicing of parenting skills, was deemed fairly relevant to relevant by 100% (27/27) of the parents. Points for improvement were also suggested, with 41% (11/27) desiring more personal email contact with facilitators and a few participants wanting telephone or face-to-face contact. Some 30% (8/27) of parents expressed dissatisfaction with the number of dropouts from the course; others valued the greater personal attention in the smaller stay-behind groups.

### Course Adherence

Of the 59 parents who began the course, 42% (25/59) took part in fewer than four sessions, and 57% (34/59) in four sessions or more; 37% (22/59) attended seven or eight sessions, and 20% (12/59) all eight sessions. The reasons reported for dropping out during the course were varied, but often involved unstable home situations (eg, relational problems and divorce, relocation, or starting a reintegration program). As reported above, completers did not significantly differ from participants who dropped out of the study on any of the measured variables, indicating that loss-to-follow up was random.

## Discussion

### Principal Results

At the onset of the study, many parents scored in the clinical range on parenting skills, indicating that they were facing serious childrearing problems. At the conclusion of the course, a large proportion of parents had moved out of the clinical range; the percentages of parents in clinical ranges for laxness and overreactivity at pretest (75% and 64%) had decreased by posttest to 43% and 39%, respectively.

Parenting skills of laxness and overreactivity (*d* = 0.52 and *d* = 0.48) decreased, parental sense of competence (*d* = 0.61) increased, and feelings of incompetence (*d* = 0.46) decreased, indicating that parents were less likely to overreact or underreact to child behavior, that parents were responding to behavior appropriately on the basis of its severity, and that parents felt more empowered in their parenting and thus less likely to generate insecure attachment styles and poor outcomes for the children [70]. The effect sizes on parenting skills and sense of competence were comparable to those seen in studies of level 4 of the parenting program Triple P.

In terms of children’s problems, the pilot results showed a decline, though not significant, on the SDQ scores. This contrasts with a significant finding for behavior problems from the Triple P level 4 program, which showed significant, moderate effects on the Eyberg Child Behavior Inventory (overall effect size *d* = 0.42) [41]. The difference in outcome may be explained by the fact that children’s problems at baseline in the Triple P study were in the clinical range, while those in our study were largely in the nonclinical range.

The fact that baseline parenting problems were largely in the clinical range and child problems in the nonclinical range suggests that parents were reached at an early stage of their parenting difficulties. The course satisfaction was high, with a mean score of 7.8 (10-point scale). The course adherence seemed to be a point for improvement and further research; 57% (34/59) followed half of the sessions or more, and only 20% (12/59) of the parents followed all the sessions. Finally, 93% (45/48) of the participants had received professional psychological help from a mental health service. This indicates a limited achievement of the aspiration to reach parents who had not been in contact with a mental health service. It is unknown, however, when these contacts took place; if this was long before the course attendance, the potential benefit of this online intervention might have been realized.

### Limitations

The most significant limitation of the study was the lack of a comparison group, making it impossible to conclude whether the significantly improved parenting competence was attributable to the course or to some other fact. The effect sizes from the trial may well have been inflated because they constituted the effects of spontaneous recovery and of nonspecific effects. It is thus likely that effect sizes in a well-controlled trial would be considerably smaller. A second limitation of this pilot study was the relatively small size. This precluded undertaking specific subanalyses, for example, to predict outcome from parent and child factors. A third limitation was the self-report nature of the quantitative parenting data. However, in keeping with the digital and anonymous nature of the intervention, independent observations were not feasible. Data on child behavior were based on reports of the parents rather than independent raters, and may have been biased. A final limitation was the lack of data from the period following the intervention so that it is not known whether the observed improvements continued, strengthened, or diminished in the longer term.

### Implications for Future Research Directions

Future research on KopOpOuders will involve a controlled trial for measuring the effects of the intervention, including longer-term effects, on parenting and on child wellbeing. Cost-effectiveness analyses will be undertaken. The costs of the online course in terms of facilitator time are about the same as costs for face-to-face courses, but these costs are lower compared with costs of individual or family counselling. The expected short-term and long-term savings of online parenting courses lie in lower costs associated with work absenteeism and eventual treatment or care for parents and children.

Because the sample included a group of parents with mixed diagnoses, further analysis will examine diagnostic, symptom, and other variables that predict outcome. Future research should also target the role of the other partner or well partner and measure well-being. Finally, another area to explore is how the target group could be better reached.

Course adherence has been found to be associated with the success of a range of mental health programs [71], yet little research has been done to analyze adherence to these programs or the factors that can improve adherence [49]. We monitored course adherence in the present study, but little other material on online parenting support is available for comparison. The study of Taylor and colleagues [48] reported on a computer-based parenting course combined with five home visits and telephone coaching. The course adherence was as follows: 66% (59/89) completed all of the program elements and 76% (68/89) completed more than half of the program. These results are more favourable than those of our study in which 20% (12/59) of the parents followed all the sessions and 57% (34/59) followed half of the sessions or more. The higher course adherence in the study by Taylor et al may be explained by different factors. First of all, in the study by Taylor et al, the participants were not anonymous and the program comprised probably fewer elements than the KopOpOuders intervention. Also, the better mental condition of the parents may be an explanatory factor. In our study, parents often cited their unstable home situation, which usually appeared linked to their mental illness, as a motive for stopping. Similarly, Dutch experiences with face-to-face courses for mentally ill parents indicate that unstable situations at home often prompt participants to drop out. Another explanatory factor that may have led to better course adherence in the study by Taylor et al is the personal contact with a counselor through home visits and telephone coaching. This corresponds to our satisfaction survey in KopOpOuders, which revealed a desire by the parents for more personal contacts with the facilitator via email, which might have strengthened participants’ commitment to the course. Future research should focus on factors that could improve course adherence and on the impact of adherence on the outcome.

### Implications for Policy and Practice

In view of the increasing numbers of children now in care [72], early preventative interventions need to be provided to at-risk groups in order to keep parenting problems from escalating. KopOpOuders.nl should fit well into low-threshold illness prevention programs. If the course is shown effective, its reach could be greatly extended by offering more courses and by intensifying recruitment efforts. One prerequisite for increasing delivery capacity is a clear funding structure for COPMI interventions as is now being developed in the Netherlands. Recruitment might be improved by advertising on relevant websites and in other media, as well as by embedding interventions like these in the continuum of youth services, thus enabling an effective referral pathway to the intervention.

The anonymity of the course was valued by the parents. This anonymity, however, does not fit with the established procedures of many health insurance companies, which require that parents are identified. A new funding structure for online services that preserve anonymity is proposed in the Netherlands. The funding structure for online interventions is now brought to the attention of several stakeholders under the Dutch Ministry of Health. We think the anonymity may be of great importance. Such anonymity may lower the barriers to seeking help and might probably help to lower the risk of child abuse. According to the Netherlands Mental Health Survey and Incidence Study of 7076 Dutch people [21], the risk of child abuse is two to three times higher for children of mentally ill parents as for other children. This includes all forms of abuse: physical, psychological, and sexual abuse, and emotional neglect. Given the clear association between child abuse and parental mental illness [20,21], and in view of these parents’ feelings of shame and their fears of losing custody of their children [25,26], the anonymity of online parenting support might breaking down barriers to their seeking help. On the other hand, what actions should course facilitators take if they suspect that child abuse is occurring? A protocol is now being drawn up by the developers of KopOpOuders to address this important issue; it will be submitted for approval to the professional sectors involved and to the Netherlands Health Care Inspectorate.

### Conclusions

Our pilot study gives reason for cautious optimism about the prevention of mental health problems in a large at-risk group—children of parents with mental illness (COPMI). The objectives of the online intervention, KopOpOuders, appear to have been nearly achieved: reaching mentally ill parents at an early stage of their parenting difficulties and enhancing their children’s well-being by improving the parents’ childrearing competence.

Future research, with a randomized controlled design, should examine the short- and long-term effectiveness of this intervention on parenting, child well-being, and the well-being of the parents. Future research should also focus on cost-effectiveness of the intervention and on course adherence and the factors that can improve it.

## Abbreviations

COPMI: children of parents with mental illness
DSM: Diagnostic and Statistical Manual of Mental Disorders
FPI: Preventive Family Intervention
OOO: Ouderlijke Opvattingen over Opvoeding (parental perceptions of parenting questionnaire)
PS: Parenting Scale
SDQ: Strengths and Difficulties Questionnaire
